# Transport and regulatory mechanisms of boron in plants

**DOI:** 10.3389/fpls.2025.1653484

**Published:** 2025-11-17

**Authors:** Dan Zhou, Rui Luo, Bojun Ma, Xifeng Chen

**Affiliations:** College of Life Sciences, Zhejiang Normal University, Jinhua, China

**Keywords:** boron, transporters, NIP, BOR, regulation

## Abstract

Boron (B) is a vital micronutrient necessary for the proper development of plants. However, B exhibits a very narrow concentration range between deficiency and toxicity in plants, making precise regulatory control over its uptake, translocation, and cellular efflux critical for maintaining overall B homeostasis. Genetic analyses of *Arabidopsis thaliana* show that boron uptake and translocation are mediated by two families of transmembrane transporter proteins: NIPs (nodulin-26-like intrinsic proteins), which facilitate the permeation of boric acid, and BORs, responsible for exporting borate from cells. Importantly, the identification and characterization of NIPs and BORs have been essential for elucidating B homeostasis and its physiological roles not only in Arabidopsis but also in diverse plant species. Furthermore, the homeostasis of B is maintained by multi-level regulation of its transport proteins, including transcriptional modulation, mRNA stability, translational repression, and endocytic degradation. Moreover, modulating B transport gene expression to enhance tolerance to B deficiency or toxicity can improve plant growth under unfavorable B nutrient conditions. Therefore, generating B-efficient or B-tolerant plants is a cost-effective and sustainable agricultural strategy. In this review, we discuss the physiological roles of B transport proteins and their regulatory mechanisms, focusing on intracellular localization and abundance.

## 1 Introduction

Nutrients are categorized as either macronutrients or micronutrients based on the quantities required for growth. These nutrients play a crucial role in regulating cellular electrochemical balance, function as biochemical cofactors, and serve as structural components within biomolecules and complexes (Baxter, 2009). Boron (B) is an essential micronutrient for normal development of plants, naturally present in the soil as boric acid (H_3_BO_3_) or borate [B(OH_4_)^-^] depending on the pH of the soil solution (Warrington, 1923; Lilay et al., 2024). Under physiological conditions, B is present primarily as boric acid in solution; boric acid is a weak Lewis acid with a pKa of 9.24, [B(OH)_3_ +H_2_O ⇋ B(OH)_4_^–^ +H^+^] (Power and Woods, 1997). Boron plays varied and complex roles in plant development, as shown by the diverse phenotypes of deficient plants. One of the primary functions of B is to facilitate the cross-linking of the pectic polysaccharide RG-II within cell walls (Kobayashi et al., 1996; Ishii and Matsunaga, 1996; Ishii et al., 1999; O’Neill et al., 2001), where over 90% of the RG-II in the plant cell wall is cross-linked by B (O’Neill et al., 2004; Matsunaga et al., 2004). In addition, it has been proposed that B serves as a component of both the plasma membrane (PM) and the cytoskeleton of the cell (Bassil et al., 2004; Voxeur and Fry, 2014).

There is a narrow range of B concentrations that supports plant growth, outside this range B can be toxic or cause deficiency symptoms. B deficiency symptoms primarily occur during plant growth, leading to inhibited expansion of young leaves, reduced root elongation, and loss of fertility (Dell and Huang, 1997; Shorrocks, 1997). On the other hand, B toxicity disrupts cellular metabolism, induces oxidative stress, promotes membrane lipid peroxidation, and triggers DNA damage, often leading to tissue necrosis (Reid et al., 2004; Sakamoto et al., 2011). Therefore, to prevent B deficiency or toxicity, plants require B transport systems in response to B levels. Since B cannot be readily re-translocated from mature to developing organs, B must be continuously absorbed from soil and transport to growing tissues in plants (Brown and Shelp, 1997). There were three distinct mechanisms reported for plants to acquire B from soil: (1) passive diffusion of uncharged boric acid under sufficient or high B availability; (2) active uptake, primarily under B-deficient conditions; and (3) facilitated diffusion mediated by channel proteins (Wimmer and Eichert, 2013). Recent findings have provided important insights into B transport in plants, along with advances in understanding its regulation. Here, we investigate B transport mechanisms, focusing on the key transporters involved, their physiological functions, and regulatory pathways.

## 2 Boron channels and transporters

### 2.1 Characterization of boron transporters

**Figure 1:**
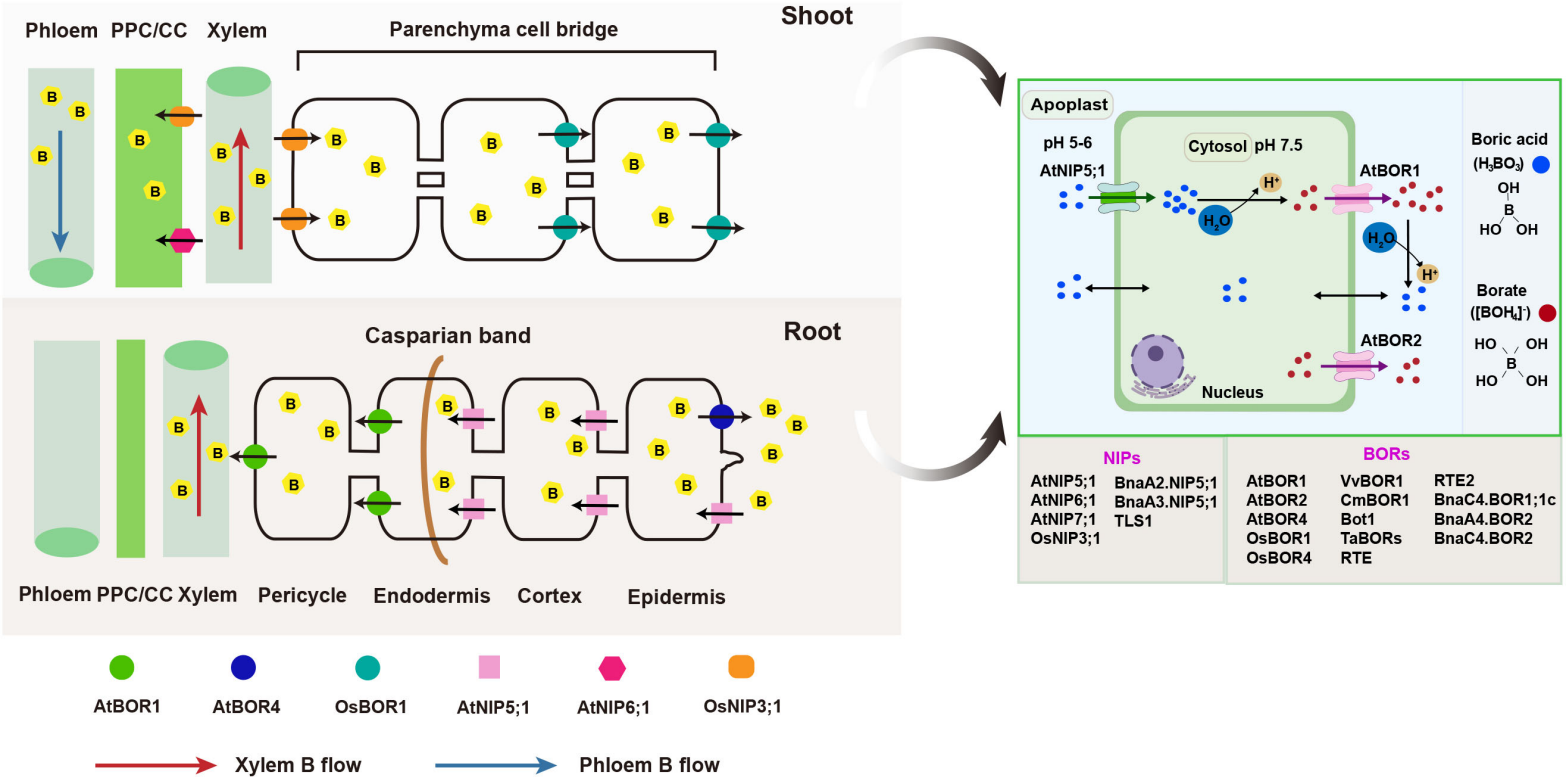
Overview of boron transporters in plants. Under low B conditions, AtNIP5;1 imports boric acid into epidermal, cortical, and endodermal cells, while AtBOR1 export boric acid/borate from stelar cells via xylem loading. Under excess B conditions, AtBOR4 enhances plant tolerance by mediating B export from roots to the soil. B is transported from roots to nodal regions via xylem, then unloaded and transferred across companion and phloem parenchyma cells to the phloem through AtNIP6;1. In rice, OsNIP3;1 is polarly localized at the xylem parenchyma cells and mediates the unloading of B from the xylem for intervascular transfer. The efflux of B for this intervascular transfer is then mediated by OsBOR1. Additionally, OsNIP3;1, located at the phloem cells, also facilitates B influx into the phloem for preferential distribution. Under physiological conditions, boric acid enters cells via specific channels. In the slightly alkaline cytosol (pH ~7.5), it is converted into borate anions and exported by borate uniporters. These anions are then reconverted to boric acid in the lower-pH (5-6) apoplast. NIPs and BORs from different plant species are listed in the colored boxes. At, *Arabidopsis thaliana*; Bna, *Brassica napus*; Cm, *Citrus macrophylla*; Os, *Oryza sativa*; PPC/CC, phloem parenchyma cells/companion cells; Ta, *Triticum aestivum*; Vv, *Vitis vinifera*.

B transport processes have traditionally been regarded as predominantly passive (Marschner, 1995). This perspective is largely due to the fact that boric acid, which is a principal form of B under physiological conditions, exists as an uncharged molecule that readily diffuses across the plasma membrane (Takano et al., 2008). However, several physiological experiments have identified active mechanisms for B transport. Dannel et al. (2000) demonstrated that B transport in sunflower (*Helianthus annuus*) occurs via carrier or channel-mediated processes. Major breakthroughs in understanding B transport mechanisms began with the identification of Arabidopsis BOR1 (AtBOR1) as the first known biological B transporter (Takano et al., 2002). Regarding the uptake and translocation of B in plants, this process is ensured by two transmembrane transporter protein families (**Figure 1**): (1) channel proteins from the NIPs (nodulin-26 like intrinsic proteins) family, which are boric acid channels that enable the passive transmembrane flow of uncharged boric acid, driven by concentration gradients; and (2) efflux transporters belonging to the BOR family, which mediate the efflux of borate ions (Miwa and Fujiwara, 2010).

### 2.2 Functions of NIPs in B transport

The molecular mechanisms of B uptake and transport in plants have been most extensively studied in Arabidopsis (Miwa and Fujiwara, 2010; Onuh and Miwa, 2021). Major intrinsic proteins (MIPs) family have been identified as boric acid channels (Wallace et al., 2006). Plant MIPs are classified into four distinct groups: the tonoplast intrinsic proteins (TIPs), the plasma membrane intrinsic proteins (PIPs), the nodulin 26 (NOD26)-like intrinsic proteins (NIPs) and the small basic intrinsic proteins (SIPs) (Wallace et al., 2006; Maurel et al., 2015). NIPs are further classified into three subclasses (I-III) based on their pore structures, and the physiological function of NIP I proteins remains unclear, while NIP II and III are known to transport boric acid and silicic acid, respectively (Wallace and Roberts, 2004; Danielson and Johanson, 2010; Roberts and Routray, 2017). In Arabidopsis, the NIP subfamily consists of nine genes (Johanson et al., 2001), including three members belonging to the NIP II subgroup: AtNIP5;1, AtNIP6;1 and AtNIP7;1 (Wallace and Roberts, 2005).

AtNIP5;1, a major boric acid channel played a crucial role in B uptake from soil under B-limited conditions (Takano et al., 2006, 2010). AtNIP5;1 has been shown to be localized on plasma membrane of lateral root cap (LRC) and epidermal cells (Takano et al., 2010). A ThrProGly (TPG) repeat in the N-terminus of AtNIP5;1 was crucial for its polar localization and effective B transport in roots (Wang et al., 2017). Expression of the *AtNIP5;1* was transcriptionally enhanced 10-fold in response to B limitation in roots (Takano et al., 2006). *AtNIP6;1* was the most similar gene to *AtNIP5;1* among the nine *NIP* genes in Arabidopsis and played a key role in the preferential translocation of B into young growing leaves (Wallace and Roberts, 2005; Tanaka et al., 2008). Limitation treatment and tracer experiments showed that B concentration were significantly reduced in young rosette leaves and shoot apices (reduced by 20% to 27%) under the conditions of B limitation in *atnip6;1* mutants, suggesting that AtNIP6;1 was required for preferential distribution of B to sink tissues (e.g., young rosette leaves, shoot apices; Tanaka et al., 2008). Both AtNIP5;1 and AtNIP6;1 were the boric acid channels on plasma membrane, and AtNIP6;1 was completely impermeable to water and involved in xylem-phloem B transfer (Takano et al., 2006; Tanaka et al., 2008). Unlike *AtNIP5;1*, B limitation resulted in a slight transcriptional upregulation (1.4-fold) of *AtNIP6;1* in stems, but no significant difference was observed in shoots (Tanaka et al., 2008). AtNIP7;1 was also identified as a boric acid channel expressed in floral anthers, functions as a water-tight boric acid permease and also transports glycerol at a lower rate (Li et al., 2011; Routray et al., 2018).

**Figure 2:**
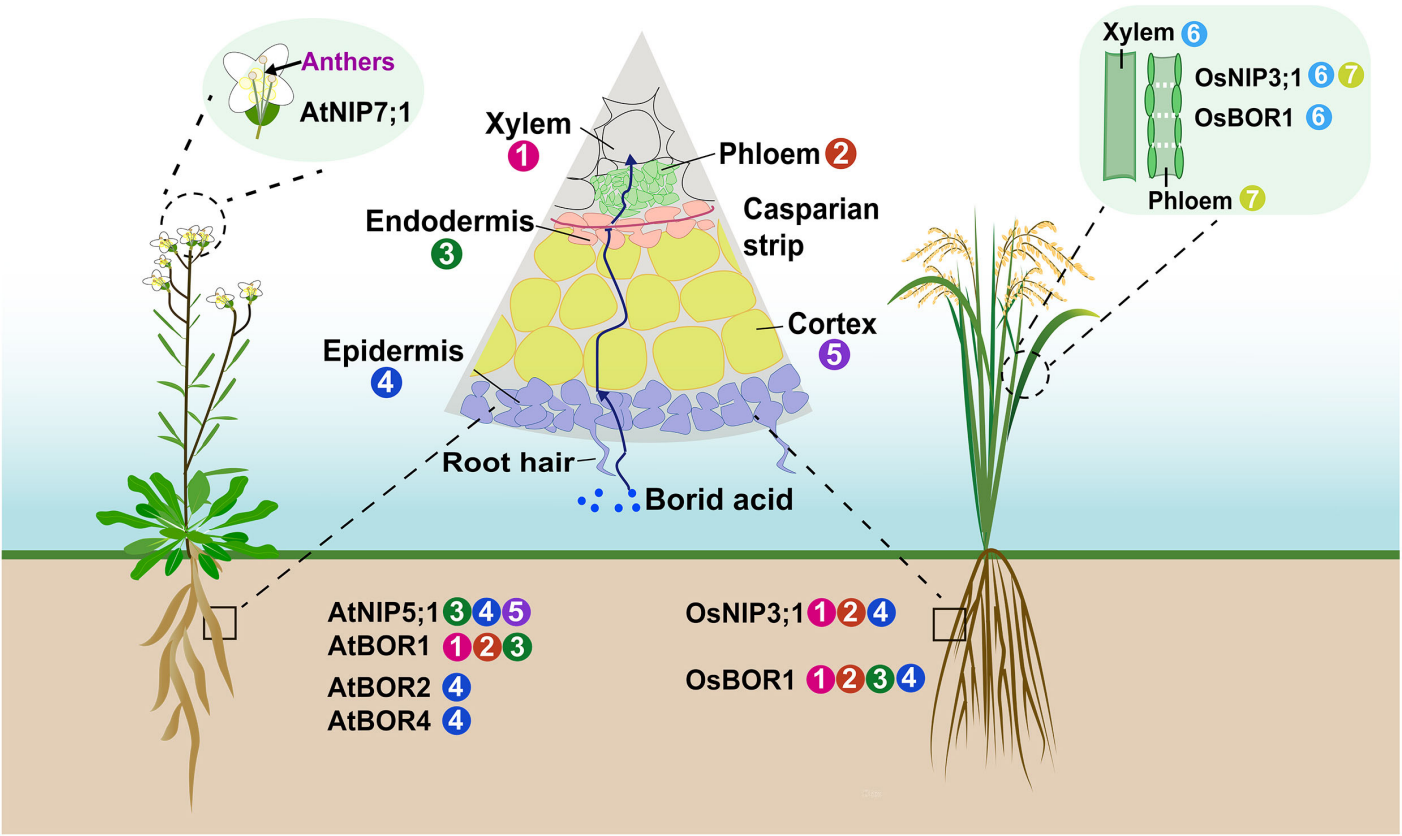
Distribution of boron transporters in various tissues. Illustration of Arabidopsis and rice plants, featuring magnifications that highlight various organs. The circled numbers adjacent to tissues indicated which transporters were predominantly expressed in each tissue. Note that not all tissues expressed these transporters.

To date, several *AtNIP5;1* homologous genes have been identified in different crops species, such as rice (*Oryza sativa*), rapeseed (*Brassica napus*) and maize (*Zea mays*) (Wallace et al., 2006; Durbak et al., 2014; Hua et al., 2016; He et al., 2021a). Rice *OsNIP3;1* exhibited the highest degree of similarity to *AtNIP5;1* (Wallace et al., 2006), and was expressed in the vascular bundles of both leaf sheaths and blades, as well as in the root exodermis and stele (**Figure 2**) (Hanaoka et al., 2014). In the nodes, OsNIP3;1 was polarly localized at the xylem parenchyma cells of enlarged vascular bundles (EVBs), facing toward the xylem vessels (Shao et al., 2018). *OsNIP3;1* RNAi plants showed disrupted B distribution between leaf blades and sheaths (Hanaoka et al., 2014). Subsequently, it was demonstrated that OsNIP3;1 mediated the unloading B from xylem of EVBs in the nodes, thus promoting its preferential distribution to developing tissues under B-limited conditions (Shao et al., 2018). TLS1/ZmNIP3;1 protein possessed the ability to transport both water and boric acid in *Xenopus laevis oocytes*, widely expressed across multiple tissue types, with highest levels in floral tissues and particularly in silks (Durbak et al., 2014; Leonard et al., 2014). Two orthologous *AtNIP5;1* genes, *BnaA2.NIP5;1* and *BnaA3.NIP5;1*, each with distinct functions, playing a crucial role in the growth of *B. napus* under B deficiency (He et al., 2021b). *BnaA2.NIP5;1* and *BnaA3.NIP5;1* functioned coordinately for efficient boron uptake. *BnaA2.NIP5;1* was primarily expressed in root epidermal cells, mediated uptake, while *BnaA3.NIP5;1* was polar-localized in the distal part of LRC cells and promoted root growth under deficiency to support translocation to the shoot (He et al., 2021a, 2021).

### 2.3 Functions of BORs in B transport

AtBOR1 was an efflux-type B transporter that expressed in pericycle cells of the root stele (**Figure 2**), functioned in xylem loading and essential for preventing B deficiency in shoots (Noguchi et al., 2000; Takano et al., 2002). Subsequently, six *AtBOR1*-homology genes were identified in Arabidopsis (Frommer and Wiren, 2002). *AtBOR2*, the most similar paralog of *AtBOR1*, functioned in root cell elongation under conditions of B limitation (Miwa et al., 2013). The concentrations of shoot B were lower in *atbor2* mutant than that in wild-type plants, but a more significant reduction was observed in *atbor1–3* under low-B conditions (Miwa et al., 2013). Thus, the role of AtBOR2 contributed to the root-to-shoot translocation was minor than that of AtBOR1 (Miwa et al., 2013). Additionally, *AtBOR4* encoded an efflux-type B transporter localized to the plasma membrane of the distal side of epidermal cells in roots and mitigated toxic levels of B in roots (Miwa et al., 2007; Miwa and Fujiwara, 2011; Miwa et al., 2014).

To date, functional *BOR* genes have been identified from different plants such as rice (*Oryza sativa*), wheat (*Triticum aestivum*), barley (*Hordeum vulgare*) and maize (*Zea mays*) (Nakagawa et al., 2007; Reid, 2007; Sutton et al., 2007; Chatterjee et al., 2014). In rice, *OsBOR1*, a close paralog of *AtBOR1*, functioned as an efflux transporter for B and played a crucial role in both the xylem loading of B and its uptake into roots (Nakagawa et al., 2007). This function, unlike AtBOR1’s exclusive role in xylem loading, resembled the combined roles of AtBOR1 and AtNIP5;1 in Arabidopsis. A recent study showed that OsBOR1 was highly expressed in the nodes, where it mediated B efflux from cells toward diffuse vascular bundles (DVBs) for delivering B to developing tissues (Shao et al., 2021). OsBOR1 cooperated with OsNIP3;1 to establish a coordinated system for the preferential distribution of B to developing tissues (Shao et al., 2021). In wheat, three functional BORs (TaBOR1.1, TaBOR1.2 and TaBOR1.3) were reported to localize on the plasma membrane in Arabidopsis leaf cells, and exhibit B efflux activity in BY-2 cells (Leaungthitikanchana et al., 2013). In maize, the B efflux transporter ROTTEN EAR (RTE) functioned as a co-ortholog of AtBOR1 and was predominantly expressed in the cells surrounding the xylem within both vegetative and reproductive tissues (Chatterjee et al., 2014); *RTE2* encoded a protein similar to its paralog *RTE* and could completely recover the deficiency of *atbor1* mutant in Arabidopsis (Chatterjee et al., 2017). RTE and RTE2 were all predominantly localized on the plasma membrane (Chatterjee et al., 2017).

*AtBOR* homologous genes have also been isolated from dicotyledonous species, such as grapevine (*Vitis vinifera*), citrus (*Citrus macrophylla*) and rapeseed (*Brassica napus*) (Pérez-Castro et al., 2012; Sun et al., 2012; Cañon et al., 2013). CmBOR1 from *C. macrophylla* and VvBOR1 from *V. vinifera* were both homologous to AtBOR1 (Cañon et al., 2013; Pérez-Castro et al., 2012). Functional assays in yeast showed that CmBOR1 mediated B efflux (Cañon et al., 2013), while VvBOR1 localized to the proximal plasma membrane of root pericycle cells and restored the wild-type phenotype in Arabidopsis *atbor1–3* mutants under B deficiency (Pérez-Castro et al., 2012). In *B. napus*, *BnaC4.BOR1;1c* was widely expressed in shoot nodes and localized to the plasma membrane, displaying characteristics generally similar to AtBOR1 (Zhang et al., 2017); Nevertheless, *BnaC4.BOR1;1c* showed distinctive features compared with *AtBOR1*, it was also extensively expressed in immature floral buds, and preferential distribution of B to the reproductive organs (Zhang et al., 2017). Two homologous genes of *AtBOR2* were identified in *B. napus*: *BnaC4.BOR2* and *BnaA4.BOR2*, both of which were mainly localized to the plasma membrane and showed B transport activity in yeast (Liu et al., 2024a, 2024). *BnaC4.BOR2*, expressed in lateral root caps and steles, was involved in B absorption in roots and its translocation to shoots (Liu et al., 2024b). *BnaA4.BOR2* was primarily expressed in the cortex and endodermis of the root tip meristem zone, as well as in the mature endodermis, it facilitated the transport of B from roots to shoots and its distribution within shoots (Liu et al., 2024a).

Conversely, while boric acid is an essential plant micronutrient, excess B inhibits plant growth, impairing various cellular functions and often causes necrosis of tissues (CamachoCristóbal et al., 2008; Landi et al., 2019; Wu et al., 2019). AtBOR4 mediated B efflux and was located on the distal surface of epidermal cells, where it reduced B concentrations in roots and shoots, thereby protecting plants from B accumulation and toxicity (Miwa et al., 2007; Takeda and Matsuoka, 2008). Unlike *AtBOR4*, *OsBOR4* in rice exhibited distinct functional characteristics, showed an anther-specific expression pattern, and was involved in maintaining boron homeostasis during fertilization (Tanaka et al., 2013). Moreover, in barley, borate exporters Bot1/HvBOR2 was responsible for the high B tolerance and protected plants from B accumulation and subsequent toxicity (Miwa et al., 2007; Sutton et al., 2007).

## 3 Physiological functions of B transporters

The roles of B in plant development seem to be diverse and intricate, as demonstrated by the variety of phenotypes observed in plants exhibiting deficiency. Depending on the growth stage or period of plant undergoing the B deficiency, their vegetative and reproductive development might be significantly or completely suppressed. In higher plants, the symptoms of B deficiency vary widely, including stunted root and shoot growth, curled and reddish leaves, aborted floral buds, reduced pod formation, and poor seed yield (Yang et al., 2013; Durbak et al., 2014).

Mutations of B transporters in plant lead to significant developmental defects (**Table 1**). *AtBOR1* was crucial for xylem loading, supporting normal shoot and reproductive development under low B conditions (Noguchi et al., 1997, 2000; Takano et al., 2002). The *atbor1–1* mutant exhibited impaired rosette leaf expansion at 3 μM B and showed normal growth but female sterility at 30 μM B, while the wild-type plants thrived under the same conditions; both defects could be fully rescued by supplementation with 100 μM B (Noguchi et al., 1997). The *atbor2–1* mutants under B deficiency exhibited impaired root cell elongation due to reduced RG-II-B dimer formation, indicating that a 50% RG-II cross-linking level was the minimum threshold for normal root elongation (Miwa et al., 2013). Moreover, the *bor1-3*/*bor2–1* double mutant displayed significantly more pronounced growth defects in both roots and shoots under B-limited conditions compared to the *bor1–3* or *bor2–1* single mutant, indicating partially redundant roles for AtBOR1 and AtBOR2 in root and shoot development under B deficiency (Miwa et al., 2013; Chatterjee et al., 2017). Similarly, growth defects in both roots and shoots were significantly diminished in loss-of-function mutants of AtNIP5;1, a boric acid channel essential for B uptake that was necessary for growth under B-limited conditions (Takano et al., 2006). Under 0.1uM B conditions, the *atnip6;1* mutant plants exhibited smaller, dark green color and irregular shape in young rosette leaves at vegetative stages, and loss of apical dominance at reproductive stages (Tanaka et al., 2008).

**Table 1:** Information and phenotypic characteristics of *NIP* and *BOR* Mutants in Plants.

Family	Plant source	Gene name	Mutants	Mutants type	B concentration	Phenotype	References
BOR	*Arabidopsis thaliana* (Arabidopsis)	*AtBOR1*	*bor1-1* *bor1-2*	EMS	Low-B (3µM)	Impaired rosette leaf expansion with reduced vegetative growth and repressed apical dominance	Noguchi et al., 1997; Takano et al., 2002, 2010
			*bor1-3*	T-DNA insertion	Sufficient-B (30µM)	Normal growth but female sterility	
					High-B (100µM)	Normal	
		*AtBOR2*	*bor2-1* *bor2-2*	T-DNA insertion	Low-B (0.1 µM)	Inhibited shoot and root growth	Kasai et al., 2011; Miwa et al., 2013
					Sufficient-B (30µM)	Normal	
		*AtBOR1/AtBOR2*	*bor1-3/bor2-1* double mutant	Single mutant hybridization	Low-B (0.1 µM)	Double mutant exhibited much more severe growth defects in both roots and shoots compared to the single mutants	Kasai et al., 2011; Miwa et al., 2013
					Sufficient-B (30µM)	Normal	
		*AtBOR4*	*bor4-1* *bor4-2* *bor4-4*	T-DNA insertion (*bor4-1*, *bor4-2*) Transposon (*bor4-4*)	Low-B (0.1 µM)	Normal	Lv et al., 2017; Miwa et al., 2014
					Sufficient-B (30µM)	Normal	
					Toxic-B (6 mM)	Reduction shoot and root growth	
	*Oryza sativa* (Rice)	*OsBOR1*	*osbor1-1* *osbor1-2*	Tos17 insertion	Low-B (0.03 µM)	Inhibited vegetative growth accompanied by sterility	Nakagawa et al., 2007
					Sufficient-B (18µM)	Normal	
		*OsBOR4*	*osbor4*	Tos17 insertion	Natural conditions	*osbor4* homozygous mutants exhibited fewer tubes and less efficient tube elongation on wild-type stigmas	Tanaka et al., 2013
	*Zea mays* (Maize)	*RTE*	*rotten ear (rte)*	EMS	Low-B (20 µM)	Exhibited stunted tassels with reduced branching and absent spikelets; leaves necrotic and wrinkled post-floral transition	Chatterjee et al., 2014, 2017
					Sufficient-B (100µM)	Restored vegetative growth and male fertility; *rte* ears developed but failed to set kernels	
					High-B (200µM)	Normal	
		*RTE2*	*rte2*	Transposon	Low-B	Significantly shorter primary roots in seedlings	Chatterjee et al., 2017
		*RTE/RTE2*	*rte;rte2* double mutant	Single mutant hybridization	Sufficient-B (0.35 ppm)	Exhibited stunted growth with chlorotic, translucent leaves; rudimentary ears; reduced root system and premature lethality after 7–8 leaves	Chatterjee et al., 2017
					High-B	Normal	
	*Brassica napus* (Rapeseed)	*BnaC4.BOR1;1c*	RNAi-1 RNAi-2	RNA interference	Low-B	Exhibited stunted growth with dark green, crimped leaves, inhibited roots at seedling stage; inhibited inflorescence with exposed stigmas, dried buds, and low seed yield	Zhang et al., 2017
					Sufficient-B	Showed abnormal flowers with stigma exsertion	
		*BnaA4.BOR2*	CR#1 CR#2	CRISPR/Cas9	Low-B (0.25 µM)	Exhibited curly dark green leaves and stunted growth; abnormal inflorescences with exposed stigmas and withered buds, leading to severely reduced seed yield	Liu et al., 2024a
					High-B (100 µM)	Normal	
		*BnaC4.BOR2*	CR#1 CR#2 CR#3	CRISPR/Cas9	Low-B	Significantly restricted growth with shorter primary roots, reduced shoot dry weight, diminished seed yield, and impaired pollen viability	Liu et al., 2024b
					High-B	Normal	
NIP	*Arabidopsis thaliana* (Arabidopsis)	*AtNIP5;1*	*nip5;1-1* *nip5;1-2*	T-DNA insertion	Low-B (3 µM/10µM)	Displayed cessation of main root growth; small rosettes; bushy stature with short internodes; defective flowers and siliques	Takano et al., 2006, 2010
					Sufficient-B (30µM)	Normal	
		*AtNIP6;1*	*nip6;1-1* *nip6;1-2* *nip6;1-3*	T-DNA Insertion	Low-B (0.1 µM/1 µM)	Exhibited darker, smaller, irregular rosette leaves and loss of apical dominance	Tanaka et al., 2008
					High-B (100 µM)	Normal	
		*AtNIP7;1*	*nip7;1-1* *nip7;1-2*	T-DNA Insertion	Low-B (0.3 µM)	Exhibited severely stunted siliques, disrupted pollen morphology, and reduced germination rates	Routray et al., 2018
					High-B (100 µM)	Normal	
	*Oryza sativa* (Rice)	*DTE1/OsNIP3;1*	*OsNIP3;1* RNAi	RNA interference	Low-B (0 µM)	Exhibited retarded growth, an increased number of tillers, and impaired pollen fertility	Hanaoka et al., 2014; Liu et al., 2015
			*dte1*	Natural selection	Sufficient-B (18 µM)	Normal	
	*Zea mays* (Maize)	*TLS1*	*tassel-less1 (tls1)*	EMS	Low-B	Exhibited a smaller SAM, progressively narrower leaves, and premature termination of growth	Durbak et al., 2014; Matthesa et al., 2018
					Natural conditions	Consistent early defects in tassel and ear development	
		*TLS1/RTE*	*tls1;rte* double mutant	Single mutant hybridization	Low-B (in Missouri )	Compared with *tls1*, not significantly enhanced in the *tls1;rte* double mutant	Leonard et al., 2014
	*Brassica napus* (Rapeseed)	*BnaA3.NIP5;1*	sRNAi*^BnaA3.NIP5;1^*	RNA interference	Low-B (0.25 µM)	Exhibited curved leaves, stubby roots, reduced biomass; ultimately fewer pods and seeds	He et al., 2021b
					High-B (100 µM)	Normal	
		*BnaA2.NIP5;1* */BnaA3.NIP5;1*	mRNAi*^BnaNIP5;1s^*	RNA interference	Low-B (0.25 µM)	Exhibited multiple branches and necrosis in the apical meristem	He et al., 2021a
					High-B (100 µM)	Normal	

EMS, Ethylmethane sulphonate.

B deficiency not only impaired vegetative growth, including inhibited root elongation and leaf expansion, but also severely disrupted reproductive development, causing early defects in the inflorescence meristem (IM) (Durbak et al., 2014). However, most studies have primarily focused on roots, with limited analysis dedicated to how these genes affect reproductive development. A higher quantity of B is required during the reproductive development phase in cereals (Shorrocks, 1997; Blevins and Lukaszewski, 1998). This increased demand may be attributed to pectin in the primary cell wall of grasses, whose content is initially low but increases throughout reproductive development (Hu et al., 1996; Matoh et al., 1996). Such an increase in pectin content impacts the key processes, including flowering, fruit set, and seed formation (Dell and Huang, 1997; Huang et al., 2000). Since B is essential for cross-linking RG-II chains, its availability in developing tissues is critical for reproductive processes like pollen germination and pollen tube growth (Dell and Huang, 1997; Blevins and Lukaszewski, 1998). *AtNIP7;1* was primarily expressed in the anthers of young flowers during a specific developmental phase, particularly at floral stages 9 and 10 (Routray et al., 2018). *AtNIP7;1* loss-of-function disrupted pollen morphology and lowered germination rates under B deficiency, indicating that AtNIP7;1 was crucial for B transport during pollen development and fertilization under low-B conditions (Routray et al., 2018).

Mutations in borate/boric acid transporters disrupt B homeostasis globally, resulting in sterile phenotypes and reproductive growth deficiencies observed in crops, including rice, maize and rapeseed. Rice and other monocot cereals have a lower boron demand than dicots due to the reduced levels of pectic compounds in their cell walls (Matoh et al., 1996). In rice, B deficiency has a more pronounced effect on reproductive growth than on vegetative growth (Uraguchi and Fujiwara, 2011). Under B-deficient conditions, *osbor1* mutants showed the sterile phenotype (Nakagawa et al., 2007). Furthermore, heterozygous *osbor4* mutants exhibited abnormal segregation ratios in their progeny, and homozygous mutants displayed defects in pollen tube germination and/or elongation, suggesting that *OsBOR4* plays a role in fertilization, a process known to require adequate boron nutrition, which is also consistent with its specific expression in anthers (Tanaka et al., 2013). The rice gene *Dwarf and Tiller-Enhancing 1* (*DTE1*), an allele of *OsNIP3;1*, was identified as the ortholog of *AtNIP5;1*, and regulates the B-dependent growth and development (Liu et al., 2015). Loss of *DTE1* function leads to vegetative and reproductive defects under low-B conditions, including growth retardation, excessive tillering and impaired pollen fertility (Liu et al., 2015). In maize, the early stages of tassel and ear development were especially sensitive to B deficiency (Durbak et al., 2014). Consistent with this notion, the maize *RTE* gene encoded a functional ortholog of the *AtBOR1* (Chatterjee et al., 2014). The *rte* mutant exhibited developmental defects in both vegetative and reproductive tissues, which impact both male and female inflorescences due to an inability to maintain activity in the inflorescence and axillary meristems (Chatterjee et al., 2014). Exogenous B application restored reproductive growth phenotypes in a dose-dependent manner (Chatterjee et al., 2014). Transmission electron microscopy (TEM) analysis of *rte* mutant ears revealed developmental-stage-dependent defects in cell wall integrity, indicating that B deficiency disrupted cell wall structure, caused expansion defects and led to cell death in meristems and floral organs (Chatterjee et al., 2014). Different from *RTE*, the disruption of *RTE2* did not affect vegetative or inflorescence development, *rte2* mutant exhibited slightly shorter roots in B-deficient conditions during early seedling growth (Chatterjee et al., 2017). However, the *rte*/*rte2* double mutant displayed more severe defects than its single mutants, showing complete growth arrest under B-deficient soils (Chatterjee et al., 2017). This B deficiency dependent phenotype was observer in poor soils but not nutrient-rich conditions, and could be fully rescued by boric acid supplementation (Chatterjee et al., 2017).

The maize *TLS1* was an allele of *ZmNIP3;1*, which predominantly expressed in floral tissues, particularly within the silks, *tassel-less1* (*tls1*) mutant displayed defects in vegetative and inflorescence development (Leonard et al., 2014). Under normal conditions, *tls1* mutants exhibited early abnormalities in tassel and ear formation (Durbak et al., 2014; Leonard et al., 2014). However, under low B conditions, they additionally showed impaired vegetative growth, characterized by a smaller shoot apical meristem (SAM), progressively narrower leaves, and premature growth termination (Durbak et al., 2014). The developmental phenotypic defects of *tls1* mutant could be rescued by application of sufficient B (Leonard et al., 2014; Durbak et al., 2014). The *tls1* mutant displayed impaired vegetative-to-reproductive transition and floral meristem development, accompanied by reduced RG-II cross-linking in immature inflorescence cell walls (Leonard et al., 2014; Durbak et al., 2014). Moreover, light intensity affected the *tls1* phenotypes: the combination of high-pressure sodium and metal halide (MH) lamps reduced the tassel phenotype severity in the *tls1* mutant under low-boron conditions by significantly increasing both transpiration and boron content (Matthesa et al., 2018).

*B. napus* is a vital oil crop with high B demand and great sensitivity to B deficiency (Xu et al., 2002). Under B deficiency, *B. napus* exhibits severe growth defects in both vegetative (inhibited root growth, leaf curling and necrosis) and reproductive (branch proliferation and stigma protrusion) organs, ultimately leading to substantial yield loss (Wang et al., 2017). *BnaC4.BOR1;1c* RNAi plants caused severe inhibition of inflorescence growth, including exposed stigma, dried-up and dropped floral buds and significantly lower seed yield (Zhang et al., 2017). Mutations in either *BnaC4.BOR2* or *BnaA4.BOR2* increased B deficiency sensitivity in *B. napus*, inhibited root growth, reduced root and shoot biomass, and severely impaired inflorescence development under low B condition (Liu et al., 2024a, 2024). These defects caused substantial yield losses, highlighting the gene’s critical role in flower organ development and seed production under low-B conditions (Liu et al., 2024a, 2024). *BnaA3.NIP5;1* RNAi plants exhibited severe developmental defects, including curved leaves and stubby roots, and caused a more than 85% decrease in seed yield per plant under low boron conditions, indicating that *BnaA3.NIP5;1* was essential for seed production in *B. napus* under boron limitation (He et al., 2021a, 2021). Compared with the *BnaA3.NIP5;1* single RNAi plants, the multiple-target knockdown lines of both *BnaA2.NIP5;1* and *BnaA3.NIP5;1* (mRNAi*^BnaNIP5;1s^*) exhibited more severe defects, such as multiple branches and apical meristem necrosis (He et al., 2021b).

In grapevine, *VvBOR1* expression level was in a stage-dependent manner during grapevine reproductive growth, with a peak in flowers at anthesis (Pérez-Castro et al., 2012). B accumulation during grapevine fruit development exhibited a biphasic pattern, peaking during the rapid growth phases (pre-veraison and post-veraison) while declining during the growth-arrested stage (Pérez-Castro et al., 2012). *VvBOR1* gene expression preceded B content increases, showing significant stage-to-stage correlation between transcriptional levels and subsequent B accumulation (Pérez-Castro et al., 2012).

## 4 Molecular mechanisms of plant responses to boron deficiency and toxicity stress

**Figure 3:**
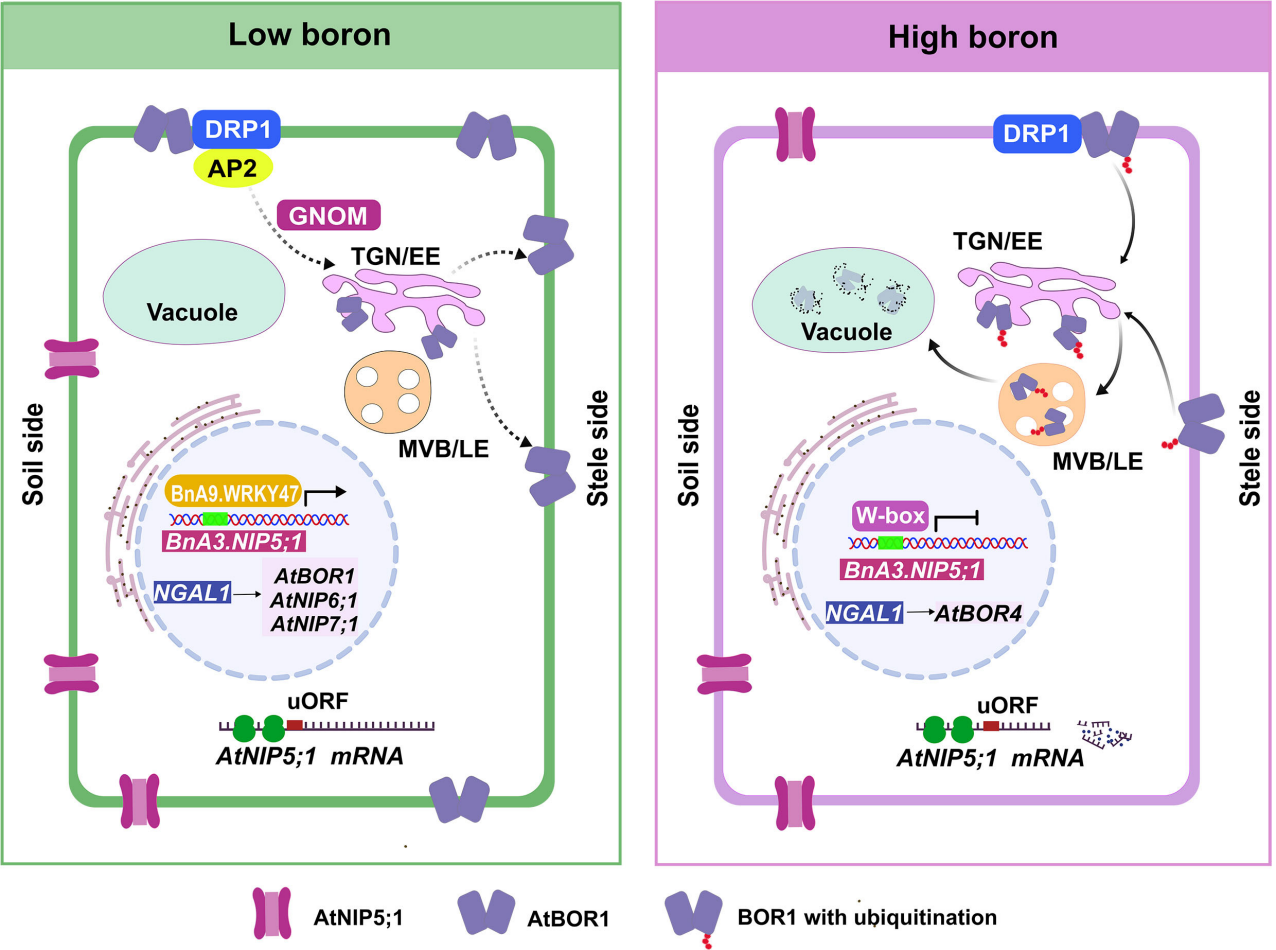
Intracellular and signaling mechanisms involved in cellular-level boron homeostasis within root cells. Under low-B conditions, AtBOR1 underwent continuous internalization from the plasma membrane into trans-Golgi network/early endosome (TGN/EE), where it was recycled back to the PM to sustain B uptake. Moreover, the transcription factor BnaA9.WRKY47 specifically activated the expression of *BnaA3.NIP5;1* by binding to the W box elements. Under high-B conditions, AtBOR1 undergoes ubiquitination, the ubiquitinated BOR1 is transported from the TGN/EE into multi-vesicular bodies/late endosomes (MVB/LE) by TOLs and endosomal sorting complex required for transport (ESCRT) machinery for vacuolar degradation, preventing excessive B transport. The expression of *BnaA3.NIP5;1* was repressed in response to boron deficiency. Additionally, ribosome stalling at AUG-stops in the 5’-UTR of *AtNIP5;1* increased under high-B conditions and was coupled with mRNA degradation. *AtNGAL1* positively regulated the expression of *AtBOR1*, *AtNIP5;1*, *AtNIP6;1* and *AtNIP7;1* in response to low B, and up-regulated *AtBOR4* in response to high B.

Due to the dual effects of B deficiency and toxicity on plant growth and development, it is important for plants to maintain B homeostasis for proper growth, and the regulation of the B transport process plays a crucial role in B homeostasis. The accumulation of B transporters is regulated by the availability of B through various regulatory mechanisms. Multiple transcriptional and post-transcriptional regulatory mechanisms have been identified to medicate acclimation to nutrient-rich (high-B) conditions (**Figure 3**). These mechanisms, regulated by B availability, ensure precise control of B uptake to prevent both toxicity and deficiency.

### 4.1 Endocytic degradation of AtBORs regulates boron levels

The mRNA levels of *AtBOR1* remained largely stable across the tested B conditions, and B translocation from roots to shoots increased under low B and decreased rapidly under high B treatment, suggesting there was post-transcriptional control of AtBOR1 (Takano et al., 2005). The trafficking of AtBOR1 shifted from PM-endosome recycling under B deficiency to endocytosis and vacuolar degradation under high B conditions, thereby regulating B homeostasis (Takano et al., 2010; Kasai et al., 2011). A series of forward studies demonstrated that DYNAMIN-RELATED PROTEIN 1A (DRP1A) and the clathrin adaptor protein ADAPTOR PROTEIN 2 (AP2)-mediated endocytosis maintained the polar localization of BOR1, thereby supporting plant growth under low-B conditions (Yoshinari et al., 2016, 2019). In contrast, boron-induced vacuolar sorting of BOR1 was DRP1-dependent but occurred through an AP2-independent endocytic pathway (Yoshinari et al., 2016, 2019). Additionally, K63-linked polyubiquitination of BOR1 at lysine 590 proved essential for its high B-induced endocytosis and degradation (Yoshinari et al., 2021a). GNOM, a guanine-nucleotide exchange factor (ARF-GEF), mediated endocytosis that contributed to maintaining BOR1 polar localization under boron-limited conditions (Yoshinari et al., 2021b). Similarly, AtBOR2, which was degraded under high B conditions, exhibited cycling behavior between the plasma membrane and endosomes under low B conditions, mirroring the dynamics of AtBOR1 (Miwa et al., 2013). In addition, OsBOR1 underwent gradual degradation in response to high B, however, its degradation pathway differs from that of AtBOR1 (Shao et al., 2021).

### 4.2 B-dependent regulation of mRNA levels

In eukaryotes, short open reading frames (ORFs) in the 5’-untranslated region (5’-UTR), known as upstream ORFs (uORFs), are often affected the translation of the downstream ORF (Jackson et al., 2010; Hellens et al., 2016). The 5’-UTR mediated B-dependent *AtNIP5;1* mRNA degradation for plant acclimation to high-B conditions (Tanaka et al., 2011). *AtNIP5;1* had two minimum ORFs (AUG-stops) in its 5’-UTR, and ribosome stalling at these AUG-stops, which was enhanced under high-B conditions and led to suppressed translation and mRNA degradation, depended on a well-conserved region 12 to 19 nucleotides upstream that acted in enhancing mRNA degradation but not in ribosome stalling (Tanaka et al., 2016). The 5’-UTRs was highly conserved between *OsNIP3;1* and *AtNIP5;1* (Tanaka et al., 2011). In rice protoplasts, the luciferase activity driven by the 5’UTR of *DTE1*/*OsNIP3;1* exhibited a dual B-dependent response, increasing at 1 μM B but decreasing at 100 μM B, indicating the 5’UTR’s essential role in B-responsive regulation and suggesting an *AtNIP5;1*-like mRNA control mechanism to prevent excessive B accumulation under high-B conditions (Liu et al., 2015).

In contrast, AtBOR1 protein abundance was regulated through two distinct mechanisms: protein endocytic degradation and B-dependent mRNA level regulation. When the B supply was sufficient (100 μM), AtBOR1 level was down-regulated by endocytic protein degradation (Takano et al., 2005; Kasai et al., 2011). However, at higher B concentrations, AtBOR1 level was decreased further by both translational suppression and protein degradation to avoidance of B toxicity in plants (Aibara et al., 2018). Furthermore, a ribosome profiling analysis revealed that transcripts with reduced translation efficiency under high-B conditions were rich in uORFs, and B played a general role in termination of translation by high B induced global ribosome stalling at the stop codon of main open reading frame (mORFs) (Sotta et al., 2021).

The abundance of B transporters in diverse plant species is coordinately controlled through B-responsive mRNA regulation. The *OsBOR1* promoter exhibited a progressive shift in its cell-specific activity between the stele and exodermis under varying B conditions, which reflected its functional adaptation to B availability (Nakagawa et al., 2007). The CTTTC tandem repeats in the *BnaA3.NIP5;1* 5’UTR negatively regulated its expression, and their deletion enhanced *BnaA3.NIP5;1* expression, which promoted root growth and increased seed yield under B limitation (He et al., 2021a). In roots, *CmBOR1* expression remained unchanged under both B deficiency and excess conditions, whereas in shoots, its expression was upregulated under B deficiency but unaffected by excess B (Cañon et al., 2013). RT-qPCR analysis of *TaBOR1s* revealed that the accumulation of *TaBOR1.1* and *TaBOR1.3* mRNA was up-regulated under B limitation, whereas *TaBOR1.2* mRNA accumulation increased under excess B conditions compared with low or normal B conditions in roots (Leaungthitikanchana et al., 2013). In contrast, *TaBORs* and *CmBOR1* exhibited distinct regulation, implying functional diversification among *BOR1* genes (Cañon et al., 2013; Leaungthitikanchana et al., 2013). This divergence may reflect species-specific adaptations, particularly in plants with complex genomes, where different *BOR1* paralogs could fulfill varied physiological roles.

Transcription factors play pivotal roles in multiple biological processes by activating or repressing the transcription of target genes (Levine and Davidson, 2005). Accumulating evidence has highlighted the importance of transcription factors in responding to nutrient conditions in plants. AtWRKY6 was the first transcription factor reported to involve in the response to B deficiency, with its promoter activity and transcription being induced by low B conditions (Kasajima et al., 2010). *BnaA9.WRKY47* positively regulated low-B tolerance through up-regulating *BnaA3.NIP5;1* expression to facilitate efficient B uptake (Feng et al., 2020). The Arabidopsis homolog *AtWRKY47* acted as a negative regulator that involved in boron homeostasis (Feng et al., 2021). *NGATHA-Like 1* (*NGAL1*, also known as *ABNORMAL SHOOT 2*, *ABS2*) was a B-responsive gene regulated in a B-dependent manner through AUG-Stop, similar to *AtNIP5;1* (Tanaka et al., 2016). *NGAL1* positively regulated the expression of *AtBOR1*, *AtNIP5;1*, *AtNIP6;1* and *AtNIP7;1* in response to low B, and up-regulated *AtBOR4* in response to high B to enhance B transport and distribution in both conditions (Tsednee et al., 2022).

## 5 Transgenic plant development to address B deficiency and toxicity

The inadequate uptake of B due to poor soil quality has emerged as a significant agricultural challenge in various regions worldwide, and crops cultivated in B-deficient soils often experience reductions in both yield and fruit quality (Shorrocks, 1997; Dell and Huang, 1997). Although B fertilizer can alleviate plant B deficiency, borate rock is a non-renewable resource. To address this problem, molecular breeding to enhance B-transporter activity represents a promising strategy for combating B deficiency in crops. On the other hand, B exhibits toxic effects when present in excessive amounts. The generation of B-deficient or tolerant plants represents a cost-effective and environmentally sustainable strategy for agriculture. There were several reports on improvement of B deficiency tolerance or toxicity by modulating expression of B channel genes to improve plant growth under unfavorable B nutrient conditions.

### 5.1 Generation of transgenic plants to mitigate B deficiency

Overexpression of *AtBOR1* enhanced root-to-shoot translocation of B, and improved shoot growth and fertility under B-deficient conditions but not root growth (Miwa et al., 2006). This was attributed to the degradation of AtBOR1 under high-B supply, and enhanced the translocation of B from root-to-shoot under low-B conditions (Takano et al., 2005; Miwa et al., 2006). Furthermore, tomato (*Solanum lycopersicum*) plants overexpressing *AtBOR1* maintained normal leaf development under B deficiency, and elevated B accumulation in shoots and fruits (Uraguchi et al., 2014). In addition, overexpression of *CmBOR1* in Arabidopsis resulted in enhanced shoot growth with limited B supply, as did overexpression of *AtBOR1* (Cañon et al., 2013). Moreover, Overexpression of *BnaC4.BOR1;1c* in the B-inefficient *B. napus* cultivar W10 alleviated shoot B-deficiency symptoms by improving boron distribution from roots to shoots (Chen et al., 2018).

AtNIP5;1 was a major boric acid channel required for efficient import of B into roots (Takano et al., 2006). Arabidopsis plants with *AtNIP5;1* activated by a T-DNA insertion with a enhancer improved root growth under B limitation, but did not improved shoot growth (Kato et al., 2009). Furthermore, introduction of *Pro*_(_*_35S+NIP5;1_*_)_*:NIP5;1* into the AtBOR1 over expressor improved root elongation, fertility and short-term B uptake under low-B supply (Kato et al., 2009). Elevated *BnaA3.NIP5;1* expression improved low-B tolerance in transgenic lines at both seedling and mature stages, and field trials demonstrated that the *BnaA3.NIP5;1^Q^* allele significantly increased seed yield under B deficiency conditions (He et al., 2021a).

### 5.2 Generation of transgenic plants to combat B toxicity

Overexpression of *AtBOR4* improved growth under conditions of B toxicity through AtBOR4-mediated B efflux that decreased B concentrations in roots and shoots (Miwa et al., 2007). *AtBOR4*-overexpressing transgenic plants were more capable of expanding leaves and accumulating chlorophyll in shoot tissues under high-B concentration, suggesting overexpressed *AtBOR4* alters B distribution in leaves by exporting B from cytoplasm into apoplasm for enhancing high-B tolerance in shoots (Miwa and Fujiwara, 2011). Arabidopsis *SHB1*/*HY1* gene, encoded HO1 (heme oxygenase 1), was up-regulated under excessive B stimulation, and the *shb1* seedlings exhibited root inhibition under excessive B treatments (Lv et al., 2017). However, overexpressing *SHB1*/*HY1* or applying the HO1 catalytic products could induced *BOR4* transcription, reduced B accumulation in roots and restored primary root growth that confers high B tolerance (Lv et al., 2017).

Moreover, in a B-stress tolerant cultivar ‘*Sahara*’ of barley, unlike intolerant genotypes, which had four tandem copies of the *Bot1* gene with higher transcript levels, and *Bot1* expression levels directly correlating with tolerance across various landraces (Hayes and Reid, 2004; Reid, 2007; Sutton et al., 2007; Mickelbart et al., 2015). Similarly, *TaBOR2* and *HvBOR2* reduced root B concentrations in the tolerant cultivars, and their expression levels showed positive correlations with tolerance (Reid, 2007; Sutton et al., 2007).

## 6 Conclusion

Boron is an essential micronutrient for plant growth. Regulating the activity of transport proteins is essential for plants to adapt to changing nutrient availability. Plants use complex homeostasis networks to regulate boron uptake, mobilization, distribution, and storage to assure proper growth. While characterizing BOR and NIP II family members has greatly advanced our understanding of boron transport systems, further research on boron transport mechanisms in cereals remains essential to optimize boron nutrient use efficiency. The regulatory mechanisms of B transport proteins include B-induced ribosome stalling and *AtNIP5;1* mRNA degradation mediated by its 5’UTR (Tanaka et al., 2011, 2016), as well as B-triggered endocytosis and degradation of AtBOR1 through its self-regulatory transceptor function (Takano et al., 2005, 2010; Yoshinari et al., 2021a). However, the involvement of additional regulatory elements or mechanisms in boron transport protein modulation remains unclear. Therefore, a systematic characterization of these proteins, including their regulatory components and interaction networks, is essential for future research. Current research on boron efficiency in plants has mainly focused on roots, leaving the mechanisms during reproductive growth poorly understood. In particular, floral organ responses to boron deficiency and their molecular regulation require urgent investigation. The development of B-deficient and B-tolerant transgenic plants by manipulating B transport proteins presents a promising strategy to reduce fertilizer use and mitigate toxicity risks. Current successes in creating plants that tolerate both low and high B levels should be optimized for crop species, promoting sustainable agriculture in areas affected by B deficiency or excess.
